# Chemo-Enzymatic Synthesis of Ester-Linked Docetaxel-Monosaccharide Conjugates as Water-Soluble Prodrugs

**DOI:** 10.3390/molecules16086769

**Published:** 2011-08-09

**Authors:** Kei Shimoda, Naoji Kubota

**Affiliations:** Department of Chemistry, Faculty of Medicine, Oita University, 1-1 Hasama-machi, Oita 879-5593, Japan

**Keywords:** docetaxel, chemo-enzymatic synthesis, glycoside, ester-linker, prodrug, cytotoxicity

## Abstract

Three new docetaxel prodrugs, *i.e.*, 7-propionyldocetaxel 3''-*O*-β-D-glycopyranosides, which contain ester-linked monosaccharides, were synthesized by a chemo-enzymatic procedure involving enzymatic transglycosylations with lactase, β-galactosidase, or β-xylosidase. The water-solubility of 7-propionyldocetaxel 3''-*O*-β-D-glucopyranoside was 52-fold higher than that of docetaxel. 7-Propionyldocetaxel 3''-*O*-β-D-glucopyranoside and 7-propionyldocetaxel 3''-*O*-β-D-xylopyranoside were effectively hydrolyzed by the relevant enzyme(s) of human cancer cells to release docetaxel, whereas 7-propionyldocetaxel 3''-*O*-β-D-galactopyranoside was relatively resistant under similar conditions. 7-Propionyldocetaxel 3''-*O*-β-D-glucopyranoside and 7-propionyldocetaxel 3''-*O*-β-D-xylopyranoside showed *in vitro* cytotoxic activity against human cancer cells, whereas 7-propionyldocetaxel 3''-*O*-β-D-galactopyranoside exerted low cytotoxicity.

## 1. Introduction

Docetaxel is a taxane diterpenoid, which shows cytotoxic activity against leukemia cells and inhibitory action against a variety of tumors [1]. It has been recognized as one of the most effective and widely used drugs for the treatment of ovarian, breast, and lung cancers. Despite its effective pharmacological activities, docetaxel has shortcomings such as low solubility in water and toxicity to normal tissues. Its prodrugs, which incorporate acids or amino acids, have attracted much attention, because ester and amide linkages improve the water-solubility of docetaxel and can be hydrolyzed to release docetaxel by hydrolytic enzymes in the living body [2,3,4,5]. However, acid or amino acid conjugates lack tumor selectivity. A number of docetaxel prodrugs have been designed and chemically prepared in order to improve drug selectivity toward tumor cells. One of the most important approaches for drug delivery is the use of saccharide based transporters [6,7,8]. Notably, saccharide conjugation drastically enhances the water-solubility of aglycones [9]. We report here the synthesis of highly water-soluble new ester-linked monosaccharide conjugates of docetaxel, *i.e.*, 7-propionyldocetaxel 3''-*O*-β-D-glycosides, and their cytotoxic activity toward human cancer cells.

## 2. Results and Discussion

### 2.1. Chemo-Enzymatic Synthesis of Docetaxel Prodrugs

**Figure 1:**
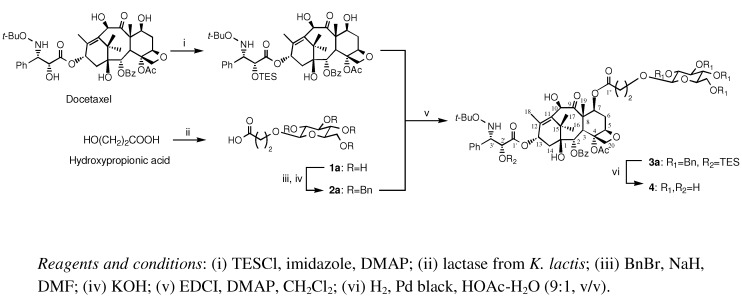
Synthesis of the docetaxel prodrug 7-propionyldocetaxel 3''-*O*-β-D-gluco-pyranoside (**4**).

7-Propionyldocetaxel 3''-*O*-β-D-glucopyranoside was synthesized by chemo-enzymatic methods, including stereoselective β-glucosylation with lactase from *K. lactis* (Figure 1). Glucosylation of hydroxypropionic acid was catalyzed by lactase from *K. lactis* and the yield of carboxyethyl β-D-glucopyranoside (**1a**) was 35%. Carboxyethyl β-D-galactopyranoside (**1b**) and carboxyethyl β-D-xylopyranoside (**1c**) were prepared in 22 and 27% yields using β-galactosidase from *A. oryzae* and β-xylosidase from *Aspergillus* sp. as biocatalysts, respectively. Carboxyethyl β-glucopyranoside (**1a**) was benzylated with BnBr/NaH in DMF at room temperature for 12 h, followed by stirring with aqueous KOH (1.5 equiv.) to give carboxyethyl 2,3,4,6-tetra-*O*-benzyl-β-D-glucopyranoside (**2a**) in 89% yield after silica gel column chromatography. The C2' hydroxyl group of docetaxel was selectively protected as a triethylsilyl ether using triethylsilyl chloride (TESCl) and imidazole in DMF affording the 2'-TES ester of docetaxel in 85% yield. The coupling of 2'-TES ester of docetaxel with **2a** (1.2 equiv.) in the presence of EDCI/DMAP in CH_2_Cl_2_ at room temperature for 12 h gave **3a** in 92% yield. Deprotection of both TES and benzyl groups with Pd black in HOAc-H_2_O (9:1, v/v) gave 7-propionyldocetaxel 3''-*O*-β-D-glucopyranoside (**4**) in 95% yield. 7-Propionyldocetaxel 3''-*O*-β-D-galactopyranoside (**5**) and 7-propionyldocetaxel 3''-*O*-β-D-xylopyranoside (**6**), were synthesized by the same method employing carboxyethyl 2,3,4,6-tetra-*O*-benzyl-β-D-galactopyranoside and carboxyethyl 2,3,4,6-tetra-*O*-benzyl-β-D-xylopyranoside in coupling reactions.

Product **4** was assigned a *Mr* of 1064.2820 [M+Na]^+^ in the HRFABMS spectrum, which suggested a molecular formula of C_5__2_H_6__7_NO_21_. In the ^13^C-NMR spectrum of **4**, the chemical shifts of the sugar carbon signals indicated that the sugar component in **4** was β-D-glucopyranose. Correlations were observed in the HMBC spectrum of **4** between the proton signal at δ 4.70 (H-1a) and the carbon signal at δ 68.0 (C-3'') and between the proton signal at δ 4.15 (H-7) and the carbon signal at δ 170.8 (C-1''). These results confirmed that the β-D-glucopyranosyl residue was attached to the hydroxyl group at C-3'' and that propionyl group was linked at C-7 of docetaxel. HMBC spectrum showed correlations between the proton signal at δ 1.75 (H-19) and the carbon signal at δ 57.7 (C-8) and between the proton signal at δ 1.08 (H-16) (δ 1.15 (H-17)) and the carbon signal at δ 44.5 (C-15). The proton signals and carbon resonances at docetaxel moiety of **4** were in good agreement with those of 7-glycolylpaclitaxel 2''-*O*-β-D-glucopyranoside [9], except for the *t*-butyloxyl group. Paclitaxel is a structural analog of docetaxel, the *t*-butyloxyl group of which is replaced by a benzoyl group. The NMR data of the *t*-butyloxyl moiety of **4** were identified by comparison with those of docetaxel [10]. Thus, compound **4** was identified as 7-propionyldocetaxel 3''-*O*-β-D-glucopyranoside.

The HRFABMS spectrum of product **5** showed a peak at *m*/*z* 1064.2815 [M+Na]^+^ suggesting a molecular formula of C_52_H_67_NO_21_. The ^13^C-NMR data of the sugar moiety of **5** agreed with those of β-D-galactopyranose. In the HMBC spectrum of **5**, correlations were observed between the proton signal at δ 4.87 (H-1a) and the carbon signal at δ 68.0 (C-3'') and between the proton signal at δ 4.15 (H-7) and the carbon signal at δ 170.8 (C-1''). These results confirmed that the β-D-galactopyranosyl residue was attached to the hydroxyl group at C-3'' and that propionyl group was linked at C-7 of docetaxel. Thus, compound **5** was identified as 7-propionyldocetaxel 3''-*O*-β-D-galactopyranoside.

Product **6** showed a pseudo molecular ion peak at *m*/*z* 1034.2770 [M+Na]^+^ (HRFABMS) consistent with a molecular formula of C_51_H_65_NO_20_. The sugar component in **6** was shown to be β-D-xylopyranose based on the chemical shifts of the sugar carbon signals. HMBC correlations between the proton signal at δ 4.70 (H-1a) and the carbon signal at δ 68.2 (C-3'') and between the proton signal at δ 4.15 (H-7) and the carbon signal at δ 170.8 (C-1'') established that the β-D-xylopyranosyl residue was attached to the hydroxyl group at C-3'' and that propionyl group was linked at C-7 of docetaxel. Thus, compound **6** was identified as 7-propionyldocetaxel 3''-*O*-β-D-xylopyranoside.

### 2.2. Water-Solubility of Docetaxel Prodrugs

The 7-propionyldocetaxel 3''-*O*-β-D-glycoside docetaxel-sugar conjugates **4**–**6** were next tested for their water-solubility. The water-solubility of compound **4** was 27 μM, which was 52-fold higher than that of docetaxel (Table 1). The water-solubility of 7-propionyldocetaxel 3''-*O*-β-D-glycosides increased in the order **6** > **5** > **4**. Among the three glycosylation derivatives the glucosyl conjugation most effectively enhanced the water-solubility of docetaxel.

**Table 1:** Water-solubility of 7-propionyldocetaxel 3''-*O*-β-D-glycosides **4**–**6**.

**Compound**	Water-solubility (μM) ^a^	Fold
**Docetaxel**	0.5	1
**4**	27.0	52
**5**	25.5	49
**6**	20.2	39

^a^ Water-solubility was measured at 25 °C.

### 2.3. Cytotoxicity of Docetaxel Prodrugs

The sensitivity of KB and MCF-7 human cancer cells to doceaxel or docetaxel-sugar conjugates **4**–**6** was examined by the MTT assay, and IC_50_ values of each test compounds are summarized in Table 2. All three docetaxel derivatives showed cytotoxicity against KB and MCF-7 human cancer cells. It has been reported that esterification with hyaluronic acid at C-7 of paclitaxel, a taxane anticancer drug, slightly reduced the cytotoxicity of paclitaxel [6,7]. Galactosyl conjugation relatively decreased cytotoxicity of docetaxel and IC_50_ value of **5** for KB cells was 41 μg/mL. The results of these experiments show that docetaxel-sugar conjugates with a monosaccharide at C-7 position would be useful water-soluble docetaxel derivatives.

**Table 2:** IC_50_ values of docetaxel and docetaxel prodrugs **4**–**6**.

**Compound**	IC_50_ (μg/mL)	
	KB cells	MCF-7 cells
**Docetaxel**	12	5
**4**	29	15
**5**	41	28
**6**	25	12

### 2.4. Release of Docetaxel from Docetaxel Prodrugs by Hydrolysis with KB Cells

Docetaxel-sugar conjugates **4**–**6** were individually incubated with KB human cancer cells. Compounds **4** and **6** were converted to docetaxel in 88 and 76%, respectively. On the other hand, a relatively low hydrolysis activity of 45% was found in the case of **5**. These findings suggest that 7-propionyldocetaxel 3''-*O*-β-D-glucopyranoside and 7-propionyldocetaxel 3''-*O*-β-D-xylopyranoside were effectively hydrolyzed by KB cells to release docetaxel and that 7-propionyldocetaxel 3''-*O*-β-D-galactopyranoside was relatively resistant against the hydrolysis by KB human cancer cells.

## 3. Experimental

### 3.1. General

Lactase from *Kluyveromyces lactis* and β-galactosidase from *Aspergillus oryzae* were gifts from Okayama University of Science. The NMR spectra were recorded in MeOH-*d*_4_ using a Varian XL-400 spectrometer (^1^H at 400 MHz, ^13^C at 100 MHz, respectively). The chemical shifts were expressed in δ(ppm) referring to tetramethylsilane. The HRFABMS spectra were measured using a JEOL MStation JMS-700 spectrometer. HPLC was carried out on a Crestpak C18S column (150 × 30 mm) [solvent: MeOH:H_2_O (2:3, v/v); detection: UV (228 nm); flow rate: 1.0 mL/min].

### 3.2. Preparation of Carboxyethyl β-D-Glycopyranosides by Enzymatic Transglycosylation

Transglycosylation using lactase from *K. lactis* as a biocatalyst to synthesis carboxyethyl β-D-glucopyranoside is as follows. To a solution of phenyl β-D-glucopyranoside (0.7 mol; a gift from Prof. Nakajima of Okayama Prefectural University), and hydroxypropionic acid (0.2 mol) in 0.1 M phosphate buffer (pH 7) was added lactase from *K. lactis* (200 U) [11]. The mixture was stirred for 12 h at 35 °C and then was extracted with *n*-butanol. The organic layer was concentrated and purified by column chromatography on silica gel to afford carboxyethyl β-D-glucopyranoside (**1a**, 0.07 mol). Lactase is also a β-galactosidase. However, lactase from *K. lactis* has been reported to be a good biocatalyst for synthesis of β-glucosides [11]. In this study, it was used for preparation of carboxyethyl β-D-glucopyranoside.

Hydroxypropionic acid (0.2 mol) was galactosylated by β-galactosidase from *A. oryzae* as follows. To a solution of phenyl β-D-galactopyranoside (0.7 mol; a gift from Prof. Nakajima of Okayama Prefectural University), and hydroxypropionic acid (0.2 mol) in 0.1 M HEPES-NaOH buffer (pH 6.0) was added β-galactosidase (1000 U) from *A. oryzae*. The mixture was stirred for 12 h at 35 °C and then was extracted with *n*-butanol. The organic layer was concentrated and purified by column chromatography on silica gel to afford carboxyethyl β-D-galactopyranoside (**1b**, 0.04 mol) [12].

The synthesis of carboxyethyl β-D-xylopyranoside was carried out as follows. To a solution containing hydroxypropionic acid (0.2 mol) and xylobiose (0.5 mol) in 25 mM of HEPES-NaOH buffer (pH 7.5) was added β-xylosidase (200 U) from *Aspergillus* sp. After stirring of the reaction mixture for 24 h at rt, the mixture was centrifuged at 3000 × gfor 10 min. The supernatant was subjected on to a Sephadex G-25 column equilibrated with water to remove the enzyme. The fractions containing glycosides were purified by preparative HPLC to give carboxyethyl β-D-xylopyranoside (**1c**, 0.05 mol) [13]. The ^1^H- and ^13^C-NMR data of **1a**–**1c** are as follows.

*Carboxyethyl* *β-D-glucopyranoside* (**1a**): ^1^H-NMR: δ 3.25–3.85 (10H, m, H-2, H-3, 2', 3', 4', 5', 6'), 4.72 (1H, d, *J* = 8.0 Hz, H-1'); ^13^C-NMR: δ 62.2 (C-6'), 68.0 (C-3), 70.4 (C-2), 72.1 (C-4'), 73.5 (C-5'), 74.1 (C-2'), 75.1 (C-3'), 100.5 (C-1') 170.0 (C-1).

*Carboxyethyl* *β-D-galactopyranoside* (**1b**): ^1^H-NMR: δ 3.20–3.88 (10H, m, H-2, H-3, 2', 3', 4', 5', 6'), 4.85 (1H, d, *J* = 7.0 Hz, H-1'); ^13^C-NMR: δ 62.9 (C-6'), 68.0 (C-3), 70.0 (C-2), 70.4 (C-4'), 72.2 (C-2'), 72.5 (C-3'), 76.7 (C-5'), 101.5 (C-1'), 170.2 (C-1).

*Carboxyethyl* *β-D-xylopyranoside* (**1c**): ^1^H-NMR: δ 3.21–3.88 (9H, m, H-2, H-3, 2', 3', 4', 5'), 4.70 (1H, d, *J* = 8.0 Hz, H-1'); ^13^C-NMR: δ 67.2 (C-5'), 68.2 (C-3), 70.4 (C-2), 72.3 (C-4'), 74.1 (C-2'), 75.1 (C-3'), 100.6 (C-1'), 170.4 (C-1).

### 3.3. Synthesis of 7-Propionyldocetaxel 3''-*O*-β-D-Glycosides

A typical procedure is described for the synthesis of 7-propionyldocetaxel 3''-*O*-β-D-glucopyranoside to exemplify the synthesis of the 7-propionyldocetaxel 3''-*O*-β-D-glycosides. To a solution of BnBr/NaH (0.15 mol) in DMF (5 mL) was added carboxyethyl β-D-glucopyranoside (**1a**, 0.03 mol). The mixture was stirred at rt for 12 h, followed by stirring with aqueous KOH (1.5 equiv.). The reaction mixture was quenched with saturated aqueous NaHCO_3_ and extracted with ethyl acetate (20 mL × 3). The ethyl acetate layer was concentrated *in vacuo* and purified by silica gel column chromatography (hexane/ethyl acetate = 2/1) to give carboxyethyl 2,3,4,6-tetra-*O*-benzyl-β-D-glucopyranoside (**2a**, 0.027 mol). To a solution of docetaxel (0.03 mol) and imidazole (0.12 mmol) in dry DMF (4 mL) was added chlorotriethylsilane (0.1 mol) dropwise at rt. The reaction mixture was stirred at rt for 2 h and diluted with ethyl acetate. The mixture was washed with water and brine, dried over MgSO_4_, and concentrated *in vacuo*. Column chromatography of the residue on silica gel (hexane/ethyl acetate = 2/1 to 1/1) gave the 2'-TES ester of docetaxel (0.025 mol). To a mixture of the 2'-TES ester of docetaxel (0.015 mol) in the presence of EDCI/DMAP (0.022 mol) in CH_2_Cl_2_ (10 mL) was added **2a** (1.2 equiv.). The mixture was stirred at rt for 12 h. The reaction mixture was extracted with ethyl acetate. The organic layer was concentrated *in vacuo* and purified by column chromatography on silica gel (hexane/ethyl acetate = 2/1) to give **3a** (0.014 mol). To a solution of Pd black (0.001 mol) in HOAc-H_2_O (9:1, v/v) was added **3a** (0.01 mol). The suspension was stirred at room temperature for 24 h. Extraction of the reaction mixture with *n*-butanol followed by column chromatography on silica gel (chloroform/methanol = 4/1) yielded 7-propionyldocetaxel 3''-*O*-β-D-glucopyranoside (**4**, 0.01 mol). HRFABMS: calcd for C_5__2_H_6__7_NO_21_Na [M+Na]^+^*m*/*z* 1064.2812, found 1064.2820; ^1^H-NMR: δ 1.08 (3H, s, H-16), 1.15 (3H, s, H-17), 1.35 (9H, s, CH_3_ in *t*-Bu), 1.75 (3H, s, H-19), 1.81 (1H, m, H-6β), 1.87 (3H, s, H-18), 2.02 (1H, dd, *J* = 15.6, 9.0 Hz, H-14a), 2.28 (1H, dd, *J* = 15.6, 9.0 Hz, H-14b), 2.37 (3H, s, CH_3_ in 4Ac), 2.58 (1H, m, H-6α), 3.20–3.88 (10H, m, H-2'', H-3'', 2a, 3a, 4a, 5a, 6a), 3.90 (1H, d, *J* = 7.2 Hz, H-3), 4.15 (1H, m, H-7), 4.18 (2H, m, H-20), 4.70 (1H, d, *J* = 8.0 Hz, H-1a), 4.79 (1H, d, *J* = 5.1 Hz, H-2'), 5.01 (1H, d, *J* = 9.0 Hz, H-5), 5.62 (2H, m, H-2, 3'), 6.15 (1H, t, *J* = 9.0 Hz, H-13), 6.21 (1H, s, H-10), 7.27 (1H, t, *J* = 7.6 Hz, *p*-H in Ph), 7.29–7.68 (6H, m, *m*-H in OBz, *o*-H in Ph,*m*-H in Ph), 7.65 (1H, t, *J* = 7.6 Hz, *p*-H in OBz), 8.10 (2H, d, *J* = 8.0 Hz, *o*-H in OBz); ^13^C-NMR: δ 10.9 (3CH_3_ in *t*-Bu), 11.3 (C-19), 14.5 (C-18), 22.1 (C-16), 22.8 (CH_3_ in 4Ac), 26.7 (C-17), 34.1 (C-6), 36.0 (C-14), 44.5 (C-3, C-15), 57.0 (C-3'), 57.7 (C-8), 62.4 (C-6a), 68.0 (C-3''), 70.1 (C-2''), 71.5 (C-7, C-13), 72.1 (C-4a), 73.6 (C-5a), 74.1 (C-2a), 74.8 (C-2'), 75.1 (C-3a), 75.5 (C-2), 76.6 (C in *t*-Bu, C-10), 77.0 (C-20), 78.8 (C-1), 81.5 (C-4), 85.1 (C-5), 100.6 (C-1a), 128.3 (*o*-C in Ph), 129.7 (*m*-C in OBz, *m*-C in Ph), 131.0 (q-C in OBz), 132.7 (*o*-C in OBz, *p*-C in Ph), 134.1 (C-11), 134.5 (q-C in Ph), 135.2 (*p*-C in OBz), 142.1 (C-12), 167.4 (C=O in OBz), 170.1 (*t*-BuOC=O), 170.8 (C-1''), 171.2 (C=O in 4Ac), 174.3 (C-1'), 203.2 (C-9).

The other two new docetaxel prodrugs, *i.e.*, 7-propionyldocetaxel 3''-*O*-β-D-galactopyranoside (**5**) and 7-propionyldocetaxel 3''-*O*-β-D-xylopyranoside (**6**), were synthesized in the same manner using the corresponding carboxyethyl β-D-glycosides. The characterization data are shown below.

*7-Propionyl docetaxel* *3''-*O*-β-D-galactopyranoside* (**5**): HRFABMS: calcd for C_5__2_H_6__7_NO_21_Na [M+Na]^+^*m*/*z* 1064.2812, found 1064.2815; ^1^H-NMR: δ 1.08 (3H, s, H-16), 1.15 (3H, s, H-17), 1.35 (9H, s, CH_3_ in *t*-Bu), 1.75 (3H, s, H-19), 1.80 (1H, m, H-6β), 1.87 (3H, s, H-18), 2.02 (1H, dd, *J* = 15.6, 9.2 Hz, H-14a), 2.28 (1H, dd, *J* = 15.6, 9.2 Hz, H-14b), 2.38 (3H, s, CH_3_ in 4Ac), 2.58 (1H, m, H-6α), 3.19-3.89 (10H, m, H-2'', H-3'', 2a, 3a, 4a, 5a, 6a), 3.90 (1H, d, *J* = 7.2 Hz, H-3), 4.15 (1H, m, H-7), 4.18 (2H, m, H-20), 4.77 (1H, d, *J* = 5.1 Hz, H-2'), 4.87 (1H, d, *J* = 7.0 Hz, H-1a), 5.01 (1H, d, *J* = 9.2 Hz, H-5), 5.60 (2H, m, H-2, 3'), 6.15 (1H, t, *J* = 9.2 Hz, H-13), 6.21 (1H, s, H-10), 7.27 (1H, t, *J* = 7.6 Hz, *p*-H in Ph), 7.30–7.68 (6H, m, *m*-H in OBz, *o*-H in Ph,*m*-H in Ph), 7.65 (1H, t, *J* = 7.6 Hz, *p*-H in OBz), 8.10 (2H, d, *J* = 8.0 Hz, *o*-H in OBz); ^13^C-NMR: δ 10.9 (3CH_3_ in *t*-Bu), 11.3 (C-19), 14.5 (C-18), 22.0 (C-16), 22.8 (CH_3_ in 4Ac), 26.7 (C-17), 34.1 (C-6), 36.0 (C-14), 44.5 (C-3, C-15), 57.0 (C-3'), 57.7 (C-8), 62.8 (C-6a), 68.0 (C-3''), 70.1 (C-2''), 70.4 (C-4a), 71.5 (C-7, C-13), 72.0 (C-2a), 72.5 (C-3a), 74.8 (C-2'), 75.5 (C-2), 76.4 (C-5a), 76.7 (C in *t*-Bu, C-10), 77.0 (C-20), 78.8 (C-1), 81.5 (C-4), 85.1 (C-5), 101.5 (C-1a), 128.3 (*o*-C in Ph), 129.7 (*m*-C in OBz, *m*-C in Ph), 131.0 (q-C in OBz), 132.8 (*o*-C in OBz, *p*-C in Ph), 134.1 (C-11), 134.5 (q-C in Ph), 135.1 (*p*-C in OBz), 142.1 (C-12), 167.4 (C=O in OBz), 170.1 (*t*-BuOC=O), 170.8 (C-1''), 171.2 (C=O in 4Ac), 174.3 (C-1'), 203.2 (C-9).

*7-Propionyl docetaxel* *3''-*O*-β-D-xylopyranoside* (**6**): HRFABMS: calcd for C_5__1_H_6__5_NO_2__0_Na [M+Na]^+^*m*/*z* 1034.2780, found 1034.2770; ^1^H-NMR: δ 1.08 (3H, s, H-16), 1.15 (3H, s, H-17), 1.35 (9H, s, CH_3_in *t*-Bu), 1.75 (3H, s, H-19), 1.80 (1H, m, H-6β), 1.87 (3H, s, H-18), 2.01 (1H, dd, *J* = 15.6, 9.0 Hz, H-14a), 2.28 (1H, dd, *J* = 15.6, 9.0 Hz, H-14b), 2.37 (3H, s, CH_3_ in 4Ac), 2.58 (1H, m, H-6α), 3.21–3.89 (9H, m, H-2'', H-3'', 2a, 3a, 4a, 5a), 3.90 (1H, d, *J* = 7.2 Hz, H-3), 4.15 (1H, m, H-7), 4.18 (2H, m, H-20), 4.70 (1H, d, *J* = 8.0 Hz, H-1a), 4.79 (1H, d, *J* = 5.1 Hz, H-2'), 5.00 (1H, d, *J* = 9.0 Hz, H-5), 5.62 (2H, m, H-2, 3'), 6.15 (1H, t, *J* = 9.0 Hz, H-13), 6.20 (1H, s, H-10), 7.27 (1H, t, *J* = 7.6 Hz, *p*-H in Ph), 7.29–7.68 (6H, m, *m*-H in OBz, *o*-H in Ph,*m*-H in Ph), 7.65 (1H, t, *J* = 7.6 Hz, *p*-H in OBz), 8.10 (2H, d, *J* = 8.0 Hz, *o*-H in OBz); ^13^C-NMR: δ 10.9 (3CH_3_ in *t*-Bu), 11.3 (C-19), 14.5 (C-18), 22.2 (C-16), 22.8 (CH_3_ in 4Ac), 26.7 (C-17), 34.1 (C-6), 36.0 (C-14), 44.5 (C-3, C-15), 57.0 (C-3'), 57.7 (C-8), 67.0 (C-5a), 68.2 (C-3''), 70.1 (C-2''), 71.5 (C-7, C-13), 72.3 (C-4a), 74.1 (C-2a), 74.8 (C-2'), 75.1 (C-3a), 75.5 (C-2), 76.6 (C in *t*-Bu, C-10), 77.0 (C-20), 78.8 (C-1), 81.5 (C-4), 85.1 (C-5), 100.6 (C-1a), 128.3 (*o*-C in Ph), 129.7 (*m*-C in OBz, *m*-C in Ph), 130.9 (q-C in OBz), 132.7 (*o*-C in OBz, *p*-C in Ph), 134.1 (C-11), 134.5 (q-C in Ph), 135.2 (*p*-C in OBz), 142.1 (C-12), 167.4 (C=O in OBz), 170.1 (*t*-BuOC=O), 170.8 (C-1''), 171.2 (C=O in 4Ac), 174.3 (C-1'), 203.2 (C-9).

### 3.4. Water-Solubility of 7-Propionyldocetaxel 3''-*O*-β-D-Glycosides

Water-solubility of 7-propionyldocetaxel 3''-*O*-β-D-glycosides was examined as follows: each compound was stirred in water for 24 h at 25 °C. The mixture was centrifuged at 100,000 g for 30 min at 25 °C. The concentration of test compounds was estimated on the basis of their peak areas using calibration curves prepared by HPLC analyses of authentic samples.

### 3.5. Cytotoxicity Assay In Vitro

The sensitivity of KB and MCF-7 cells to docetaxel or **4**–**6** was determined according to the previously reported method [10]. Cells were diluted with culture medium to the seeding density (10^5^ cells/mL), suspended in 96-well tissue culture plates (100 μL/well), preincubated at 37 °C for 4 h, and then treated for 24 h with docetaxel or **4**–**6** at various concentrations to obtain a dose–response curve for each compound. After incubation, 20 μL MTT solution (2.5 mg/mL) was added to each well and the plates were further incubated for 4 h. Absorbance at 570 nm was measured with a microplate reader model 450 (BIO-RAD). Dose-response curves were plotted on a semi-log scale as percentage of the cell numbers in control cultures not exposed to test compounds.

### 3.6. Hydrolysis of 7-Propionyldocetaxel 3''-*O*-β-D-Glycosides by KB Cells

To a 5-mL vial containing RPMI 1640 medium (1 mL, Nissui Pharmaceutical Co. Ltd.) and KB cells (20 mg) was added 5 μmol of each compound. The mixture was incubated at 37 °C for 24 h. The cells and medium were separated by centrifugation at 10,000 g for 5 min. The cells were extracted with MeOH. MeOH extract was concentrated, and the residue was partitioned between H_2_O and CH_2_Cl_2_. The medium was extracted with CH_2_Cl_2_. The CH_2_Cl_2_ fractions were combined, concentrated, and analyzed by HPLC. The yield of docetaxel was calculated on the basis of the peak area from HPLC using a calibration curve provided by HPLC analyses of authentic docetaxel.

## 4. Conclusions

Three new docetaxel prodrugs were successfully synthesized by the chemo-enzymatic procedures. The glycosyl derivatives (carboxyethyl) of glucose, galactose, xylose are prepared through enzymatic glycosylation. The intermediates are useful for the preparation of docetaxel-monosaccharide conjugates. These docetaxel prodrugs were effectively hydrolyzed by the relevan enzymes of human cancer cells releasing docetaxel. The present chemo-enzymatic synthesis is useful for the practical preparation of docetaxel prodrugs. Studies on the antitumor activity *in vivo* of the prodrugs prepared are now in progress.
